# Magnetoencephalography Imaging Reveals Abnormal Information Flow in Temporal Lobe Epilepsy

**DOI:** 10.1089/brain.2020.0989

**Published:** 2022-05-12

**Authors:** Kiwamu Kudo, Hirofumi Morise, Kamalini G. Ranasinghe, Danielle Mizuiri, Abhishek S. Bhutada, Jessie Chen, Anne Findlay, Heidi E. Kirsch, Srikantan S. Nagarajan

**Affiliations:** ^1^ Biomagnetic Imaging Laboratory, Department of Radiology and Biomedical Imaging, University of California, San Francisco, San Francisco, California, USA.; ^2^ Medical Imaging Business Center, Ricoh Company Ltd., Kanazawa, Japan.; ^3^ Memory and Aging Center, Department of Neurology, University of California, San Francisco, San Francisco, California, USA.; ^4^ Epilepsy Center, Department of Neurology, University of California, San Francisco, San Francisco, California, USA.

**Keywords:** atlas-based connectivity, epilepsy, magnetoencephalography, phase-transfer entropy

## Abstract

**Background/Introduction:**

Widespread network disruption has been hypothesized to be an important predictor of outcomes in patients with refractory temporal lobe epilepsy (TLE). Most studies examining functional network disruption in epilepsy have largely focused on the symmetric bidirectional metrics of the strength of network connections. However, a more complete description of network dysfunction impacts in epilepsy requires an investigation of the potentially more sensitive directional metrics of information flow.

**Methods:**

This study describes a whole-brain magnetoencephalography-imaging approach to examine resting-state directional information flow networks, quantified by phase-transfer entropy (PTE), in patients with TLE compared with healthy controls (HCs). Associations between PTE and clinical characteristics of epilepsy syndrome are also investigated.

**Results:**

Deficits of information flow were specific to alpha-band frequencies. In alpha band, while HCs exhibit a clear posterior-to-anterior directionality of information flow, in patients with TLE, this pattern of regional information outflow and inflow was significantly altered in the frontal and occipital regions. The changes in information flow within the alpha band in selected brain regions were correlated with interictal spike frequency and duration of epilepsy.

**Conclusions:**

Impaired information flow is an important dimension of network dysfunction associated with the pathophysiological mechanisms of TLE.

**Impact statement:**

A complete description of network dysfunction in temporal lobe epilepsy (TLE) requires investigation of the flow of information in brain networks. In this whole-brain magnetoencephalography imaging study, using an information theory measure, phase-transfer entropy, we examine the strength and directionality of information flow in patients with TLE. Abnormal information flow was documented and was associated with clinical measures related to epilepsy in certain brain regions. The approach described here may provide a useful framework to investigate network dysfunction in other neurological and psychiatric disorders.

## Introduction

Among people with epilepsy that is refractory to treatment with medications, those who have seizures arising from a focal region may have curative or palliative treatment directed at that region using surgery or neurostimulation. Traditionally, the origin of seizures in focal epilepsy was considered to be anatomically isolated. More recently, neuroscience has transformed our understanding of brain networks, and the classic concept of “epileptogenic zone” has been replaced by that of “ictal network” (Zijlmans et al., 2019). Deciphering the relationship of pathophysiological mechanisms of epilepsy on the functional anatomy of brain networks provides a direct link to probe the seizure semiology—the clinical manifestation of the spread of ictal activity in the brain. A reliable, quantifiable, and objective biomarker of network disruption in people with focal epilepsy versus those without neurologic disease could serve as a key measure, therefore in delineating regions of interest for further inquiry and in providing a metric for correlative analyses.

Technological developments in noninvasive neuroimaging combined with powerful network modeling tools have opened new opportunities to characterize and quantify the patterns of network disruption in epilepsy more accurately than before. Indeed, widespread network disruption has been reliably demonstrated in patients with epilepsy and has been identified as an important contributor to cognitive deficits as well as a predictor of surgical outcome (Bonilha et al., 2015; Englot et al., 2015; He et al., 2017; Martire et al., 2020; Morgan et al., 2017; Pressl et al., 2019). Current methods examining functional network disruption in epilepsy are largely focused on the strength of network connections, inferred from statistical dependencies between the time series of neuronal activity at different brain regions. A complete description of network dysfunction in focal epilepsy, however, requires an investigation of the flow of information as well as the strength of network connections. Recent neuroimaging studies using magnetoencephalography (MEG) have reliably used statistical measures of information flow such as phase-transfer entropy (PTE) and directional PTE (dPTE) on beamformer-reconstructed time series data of neuronal activation to demonstrate typical information flow in resting-state brain networks (Hillebrand et al., 2016). How focal epilepsy affects this information flow in the human brain remains unknown, and identification of such patterns will further refine network biomarkers of seizure semiology, of seizure onset zone, and of patterns of cognitive dysfunction in people with epilepsy.

A handful of studies have used electroencephalography (EEG) to study network information flow in patients with a common type of focal epilepsy, temporal lobe epilepsy (TLE); because it is generally believed (but not proven; see, e.g., Wiebe, 2000) to be relatively heterogenous and prevalent, TLE has been the common choice for such studies. Several other studies have applied entropy metrics to locate the seizure onset zone in patients with TLE with promising results. A combined PTE and graph theory approach on EEG sensor data identified the EEG sensors closest to the seizure onset and provided a quantitative measure to localize the epileptogenic zone in patients with TLE (Wang et al., 2017). Using intracranial EEG electrodes, an analysis utilizing a symbolic transfer entropy (TE) measure was able to reliably identify the hemisphere containing the seizure onset zone without observing ictal activity (Staniek and Lehnertz, 2008). These studies indicate that TE calculated on data obtained during the interictal period is a sensitive index to quantify the functional integrity of the seizure network in TLE. However, no study has examined large-scale information flow changes across brain networks in TLE using noninvasive reconstructions of brain activity.

The transfer-entropy measures, such as PTE, are categorized into entropic measures. According to Robinson and colleagues (2013), such measures derive estimates of information computations that are not seen in power and energy estimates and have the ability to uncover a hidden dynamical structure in turbulent or chaotic dynamics in resting state. It can therefore be expected that TLE-specific turbulent dynamics, which may exist even in interictal resting state as a form of the spread of ictal activity in the brain, would be captured by using entropic measures.

In the current study, we used MEG-imaging (MEGI), with its millisecond precise temporal resolution and superior spatial resolution compared with EEG, to investigate abnormalities in information flow, choosing as our population a group of patients with medically refractory TLE who were surgical candidates.

Specifically, using whole-brain activity reconstructions, we quantified PTE and dPTE in the alpha (8–12 Hz) and delta/theta (1.5–7.5 Hz) frequency bands in a cohort of patients with TLE and in a cohort of age-matched healthy controls (HCs). Furthermore, we examined the associations of PTE and dPTE with clinical measures, including spike frequency and duration of epilepsy. We tested the hypotheses (1) that patients with TLE will exhibit frequency-specific deficits in measures of information flow compared with age-matched healthy participants and (2) that these deficits will be correlated with clinical measures related to epilepsy.

## Materials and Methods

### Subjects

Nineteen study subjects with TLE were selected from patients referred for MEG as part of a presurgical clinical epilepsy evaluation at the University of California, San Francisco (UCSF) Biomagnetic Imaging Laboratory (BIL) between June 1, 2004, and July 18, 2018, and who then underwent surgical resection for medically refractory epilepsy following the MEG recordings. Twelve out of these 19 subjects were previously included in a published work from the laboratory regarding connectivity (Englot et al., 2015). For comparison, 20 control subjects aged younger than 56 years and with no known history of seizure or neurological disorder were included for analysis; their studies were recorded during the same period. All procedures were carried out in full compliance with the UCSF clinical research policies and with the approval of the UCSF Committee on Human Research. This study was approved by the UCSF Institutional Review Board (IRB).

### Data acquisition and preprocessing

Each subject underwent MEG recording inside a magnetically shielded room with a 275-channel whole-head axial gradiometer system (MEG International Services Ltd., Coquitlam, British Columbia, Canada). Ten- to 40-min resting-state MEG recording was collected from each subject, while lying supine with eyes closed (sampling rate, 600 Hz or 1 kHz). This study protocol required the participants to be awake as much as possible during the epoch of interest.

As the first step of data preprocessing, all the subjects' data were downsampled to 600 Hz, and cardiac and blink artifacts were removed using an independent component analysis (Ablin et al., 2018). The recorded sensor time series were then segmented into 12-sec-duration epochs. Noisy epochs, for example, those containing artifact caused by head or body motion, were removed based on visual inspection of data. In addition, an automatic artifact rejection tool as implemented in the Fieldtrip toolbox (Oostenveld et al., 2011) was used to remove the epochs with muscle artifact. From the remaining clean epochs, 10 epochs were chosen for analysis, that is, total 120-sec resting-state sensor time series were used for analysis for each subject. After these artifact rejection procedures, the sensor time series were filtered using a 1–55 Hz bandpass filter. Spike frequency was calculated using the clinical annotations of “spike” in the resting-state sensor signal (10–40 min) for each subject as marked by a certified MEG-EEG technologist and reviewed by a clinical neurophysiologist and epileptologist magnetoencephalographer (H.E.K.) before inclusion.

To provide anatomical head models for MEG analysis, a high-resolution 3D T1-weighted whole-brain volumetric magnetic resonance imaging (MRI) was acquired for each subject using a 3T scanner (Excite; GE). For all participants, the outline of the brain on the structural scans was extracted, and the segmented brain was treated as a volume conductor model for the source reconstruction described below. Coregistration of the MEG data with the structural MRI was performed based on three fiducial coil positions (nasion and left and right preauricular). The result of the coregistration was confirmed by visual inspection. The signal preprocessing and coregistrations were performed using the Fieldtrip toolbox in MATLAB (Oostenveld et al., 2011).

### Source reconstruction

For source reconstruction, 6-mm regular voxels were generated in the brain region of a template MRI, “HCP40 MNI 1.25mm.nii,” that was derived from 40 HCP subjects in MNI152 space (Fan et al., 2016) resulting in 8191 voxels. The generated voxels were warped into individual head model, and then individual magnetic lead field vectors were computed on each voxel as a forward model using a single-shell model approximation (Nolte, 2003). The voxels for each subject were indexed to the Brainnetome atlas (Fan et al., 2016). The Brainnetome atlas is composed of 210 cortical regions and 36 subcortical regions. In our analyses, among these total 246 brain regions, we focused on the 210 cortical brain regions where MEG source reconstructions are considered to work well. These cortical regions, and their abbreviations used in this study, have been listed in the Supplementary Materials (Supplementary Tables S1 and S2). Montreal Neurological Institute (MNI) coordinates of the regions are also presented in the tables.

An array-gain scalar beamforming method (Sekihara et al., 2004) was applied to the 120-sec sensor time series to obtain source-localized activity for all brain regions. Beamformer weights were calculated in the time domain, and the data covariance matrix for beamforming was computed using the whole 120-sec time series; singular value truncation with 220 components was used when inverting the covariance matrix. This beamforming provided voxel-level source timecourses on the 6-mm volumetric grids in the brain. Using the Brainnetome atlas (Fan et al., 2016), 210 cortical region representative source timecourses were extracted: for each cortical region, the voxel-level source timecourse that had the maximum power was designated as the region-of-interest (ROI) representative (Hillebrand et al., 2012). The 210 ROI-level representative source timecourses were digitally filtered using a Fourier transform into delta/theta (1.5–7.5 Hz), alpha (8–12 Hz), and beta (12–30 Hz) frequency bands before computing connectivity metrics, resulting in frequency-specific ROI-level timecourses ${\left\{s_{i}\left(t;f\right)\right\}}_{i=1,\ldots ,210}$ (*t* denotes time points, and i is the label for ROI) for each frequency band (*f* denotes a label for the frequency bands, i.e., f= delta/theta, alpha, or beta). Note that for our analyses, the delta (1.5–4 Hz) and theta (4–7.5 Hz) frequency bands were grouped together into one larger band, the delta/theta band, because the analysis results did not differ substantially between these bands in an exploratory analysis. Computation of source reconstructions and connectivity metrics described above were performed using the custom-made MATLAB code.

### Phase-transfer entropy

PTE (Lobier et al., 2014) was used to evaluate pairwise directional interactions between ROI timecourses, and is considered to represent the information flow between the ROIs based on phase timecourses, ${\left\{\theta_{i}\left(t;f\right)\right\}}_{i=1,\ldots ,210},$ that were derived using the Hilbert transform of the bandpass-filtered ROI-level timecourses, ${\left\{s_{i}\left(t;f\right)\right\}}_{i=1,\ldots ,210}$. PTE is an extension of the TE measure (Bossomaier et al., 2016; Vicente et al., 2011) that was originally proposed by Schreiber (2000): 
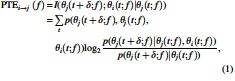


where δ is a delay, $p\left(\cdot ,\cdot \right)$ is joint probability, p(⋅|⋅) is conditional probability, and I(Y;X|Z) represents conditional mutual information between *Y* and *X* conditioned with *Z*. In the Expression (1), *i* denotes an ROI that plays a role in sending phase information, that is, a “sender,” and *j* denotes an ROI that plays a role in receiving phase information, that is, a “receiver.” According to Lobier and colleagues (2014), PTE is more robust than original real-valued TE as a measure of network connectivity.

To evaluate PTE, we used a binning method with the number of bins, *N*_bin_, set to 15. Phase [0,2*π*) is accordingly discretized into 15 bins of equal width, reducing continuous state space. This binning step thus serves to represent phase in a simpler, symbolic way (Staniek and Lehnertz, 2008), and the calculation of PTE is therefore computationally efficient. To evaluate the joint and conditional probabilities included in the definition [Eq. (1)], the symbolized phase timecourses for all 10 epochs were used in computations for each subject.

For the delay δ, we chose $\left(\delta =\right)5f_{s}^{-1}∼8.33ms,$ where $f_{s}\left(=600Hz\right)$ is the sampling frequency, although we acknowledge that PTE depends on the choice of δ (Lobier et al., 2014). The reason for the choice is as follows: the typical delay associated with the conduction of neuronal activity from various brain areas to the corpus callosum in humans is typically 5–10 ms (Terasaki and Okazaki, 2002; Tomasi et al., 2012). Our chosen delay parameter of ∼8.3 ms corresponds to this typical delay magnitude. PTE, given the above choice of *δ*, may therefore capture the effect of a sender *i* at one side of the brain on a receiver *j* located in the opposite hemisphere of the brain if there is in fact information flow between them; we may thus consider this choice suitable for querying pairwise information flow in the whole brain. In addition, this value of δ is almost the same as that used in Hillebrand and colleagues (2016) and Engels and colleagues (2017), especially for the alpha band signal with about 10 Hz, and so, we believe this choice to be justified.

It is known that TE estimates are generally biased, especially for small-sample sizes (Marschinski and Kantz, 2002), that is, TE may have a nonzero value even if there is no causal link. This is also the case for PTE calculations, and so, a bias correction is needed (Gourévitch and Eggermont, 2007). To remove bias from PTE_*i*→*j*_, an estimate of PTE is also made for shuffled data, ${PTE}_{i\to j}^{shuffled}\left(f\right)=I\left(\theta_{j}\left(t+\delta ;f\right);\theta_{i}^{shuffled}\left(t;f\right)|\theta_{j}\left(t;f\right)\right)$, and then this is subtracted from $PTE_{i\to j}\left(f\right)$ (Gourévitch and Eggermont, 2007). Here $\theta_{i}^{shuffled}\left(t;f\right)$ is a direct shuffle of *i*th ROI phase timecourse. This shuffle estimate was repeated 10 times for each $\theta_{i}\left(t;f\right)$ by randomizing the order of time points, and ${PTE}_{i\to j}^{shuffled}\left(f\right)$ was evaluated as an average of results obtained from the 10 trials. In addition to this bias correction, following Gourévitch and Eggermont (2007), we introduced normalization and defined a normalized PTE (NPTE) as

(2) $$NPTE_{i\to j}\left(f\right)=\frac{PTE_{i\to j}\left(f\right)-{PTE}_{i\to j}^{shuffled}\left(f\right)}{H\left(\theta_{j}\left(t+\delta ;f\right)|\theta_{j}\left(t;f\right)\right)}\in \left[0,1\right],$$

where H(Y|Z) is the Shannon entropy of *Y* conditioned with *Z*. This represents the fraction of information in *j*th ROI phase timecourse that cannot be explained by its own past, but can be explained by the past of *i*th ROI phase timecourse.

By computing $NPTE_{i\to j}\left(f\right)$ for all pairwise cortical ROIs, a matrix form of NPTE, which has $N_{ROI}\times N_{ROI}$ dimensions [$N_{ROI}\left(=210\right)$: the number of Brainnetome ROIs on the cortical region], is obtained. A vector-form regional measure, regional NPTE, can be defined by averaging over the components of the NPTE matrix along receiver $\left(j\right)$ array dimension or sender $\left(i\right)$ array dimension. Specifically, the regional NPTEs were defined as follows:

(3) $$NPTE_{out}\left(i;f\right)=\frac{1}{N_{ROI}-1}\sum_{j\left(\neq i\right)}NPTE_{i\to j}\left(f\right)$$

and

(4) $$NPTE_{in}\left(j;f\right)=\frac{1}{N_{ROI}-1}\sum_{i\left(\neq j\right)}NPTE_{i\to j}\left(f\right).$$

$NPTE_{out}\left(i;f\right)$, which results from averaging along receiver $\left(j\right)$ array, denotes regional information outflow at a brain region *i*, and $NPTE_{in}\left(j;f\right)$, which results from averaging along sender $\left(i\right)$ array, denotes regional information inflow at a brain region *j*. Regional NPTE corresponds to “node strength” in the NPTE network in graph theory terminology (Sporns, 2010).

In addition, we defined dPTE, which quantifies the relative preferred direction of information flow, by

(5) $$dPTE_{i\to j}\left(f\right)=\frac{NPTE_{i\to j}\left(f\right)-NPTE_{j\to i}\left(f\right)}{NPTE_{i\to j}\left(f\right)+NPTE_{j\to i}\left(f\right)}\in \left[-1,1\right].$$

In this definition of directionality, $dPTE_{i\to j}\left(f\right)=0$ in the case of no preferred direction. The form of the Expression (5) is analogous to the laterality index [or laterality quotient, e.g., Eq. (1) in Oldfield (1971)] that is often used in neuroimaging research to assess brain hemispheric dominance.

Regional dPTE was also defined, considering a dPTE matrix is antisymmetric, as follows:

(6) $$dPTE\left(i;f\right)=\frac{1}{N_{ROI}-1}\sum_{j\left(\neq i\right)}dPTE_{i\to j}\left(f\right).$$

This denotes the likelihood of the *i*th ROI being an information sender on average in comparison with other ROIs, and thus, a negative $dPTE\left(i;f\right)$ denotes the likelihood of the *i*th ROI being an information receiver on average. For visualizing these regional metrics, the BrainNet Viewer toolbox (Xia et al., 2013) was used.

### Statistics

To evaluate deviation in directional information flow, including global, regional, or matrix measures, in people with TLE, we compute *Z* scores for each patient relative to the measure's mean and standard deviation in the HC group, weighted by its sign:

(7) $$Z_{k}=\frac{w_{k}-{\bar{w}}^{HC}}{\sigma^{HC}}\cdot sgn\left({\bar{w}}^{HC}\right),$$

where *w_k_* is the value of the connectivity metric, representing global, regional, or matrix directional information flow, $k\left(=1,\ldots ,19\right)$ denotes the study subject label of TLE patients, ${\bar{w}}^{HC}$ denotes an average of the metrics $w^{HC}$s, and $\sigma^{HC}$ denotes the standard deviation of $w^{HC}$s. The sign function allows us to identify that a negative (positive) *Z_k_* indicates the decrease (increase) for NPTE or dPTE. We also performed correlation analysis with the spike frequency and duration of epilepsy, as well as estimations of the differences between groups, HC and TLE, in the connectivity metrics.

We tested for demographic differences between the TLE and HC groups, including age, using the unpaired Student's *t*-test, and gender difference using the $\chi^{2}$ test.

To compare group-level $N_{ROI}\times N_{ROI}$ NPTE or dPTE matrices between TLE and HC groups, we used one-sample *t*-tests for *Z_k_*s and obtained *p* values that were then corrected by the false discovery rate (FDR) (Benjamini and Hochberg, 1995). The FDR-corrected *p* values were defined as significant at *p* < 0.001 for NPTE and *p* < 0.01 for dPTE matrices, respectively. Since the dPTE matrix is antisymmetric (Supplementary Materials section 4), only the components belonging to its upper triangle, that is, $N_{ROI}\times \left(N_{ROI}-1\right)∕2$ components, are considered for the FDR correction.

To compare group-level regional NPTE or dPTE values between TLE and HC, we used nonparametric permutation tests (Nichols and Holmes, 2002) for *Z_k_*s and obtained *p* values. As discussed in detail in Eklund and colleagues (2015), a nonparametric permutation test is based on a small number of assumptions and has here been proven to yield more accurate results than parametric methods. The drawback of a permutation test is the increase in computational complexity. For example, a group analysis needs to be repeated 1000–10,000 times. In our analysis, the number of the permutations was set at 10,000. As before, the *p* values were corrected by the FDR, and the FDR-corrected *p* values were defined to be significant at *p* < 0.05.

For the correlation analyses, Pearson's correlation coefficients, *r*, were calculated between regional *Z_k_*s and the spike frequency and between regional *Z_k_*s and the duration of epilepsy, respectively. Permutation tests were used to evaluate *r* and the corresponding *p* values were obtained. The *p* values were corrected by the FDR, and the FDR-corrected *p* values were defined to be significant at *p* < 0.05.

In Supplementary Materials (section 5), we have shown results of the similar analyses done using a different connectivity metric, imaginary coherence (ImCoh), for reference. The same statistical methods described above for dPTE were used for the ImCoh matrix and its regional values.

## Results

### Participant characteristics

Table 1 summarizes participant characteristics in this study. Age [*t*(37) = −1.82, *p* = 0.0776] and gender [*χ*^2^(1,39) = 1.242, *p* = 0.265] did not differ between groups.

**Table 1.** Participant Characteristics

	TLE	HC	p
*n*	19	20	—
Age, years	33.3 (10.4)	39.2 (9.8)	0.078
Gender, F/M	9/10	6/14	0.265
Duration of epilepsy, years	19.3 (13.2)	N/A	—
Spike frequency, 1/min	1.2 (1.0)	N/A	—

*n* is the number of participants of each group. Values for age, duration of epilepsy, and spike frequency indicate means, and values in parentheses denote standard deviations.

HC, healthy control; TLE, temporal lobe epilepsy.

### Spectral characteristics

**FIG. 1.**
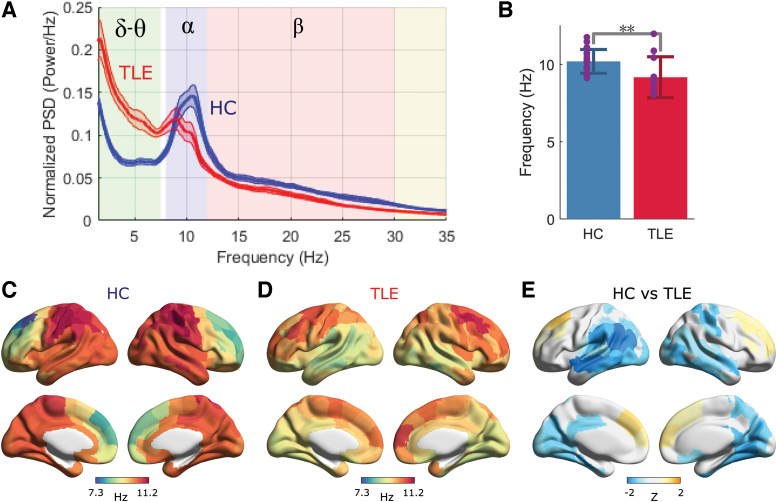
**(A)** Normalized PSDs of source timecourses averaged over the whole brain in each subject group: red for TLE (*n* = 19) and blue for HCs (*n* = 20). Frequency ranges, *δ*–*θ* (1.5–7.5 Hz), *α* (8–12 Hz), and *β* (12–30 Hz), are highlighted in different colors. Shaded zone around each PSD line depicts standard error. **(B)** Alpha peak amplitude distributions (8–12 Hz) in the normalized PSD for HC and TLE. Alpha peak in TLE shifts leftward in comparison with HC (*t*-test, **p = 0.0065 [*<*0.01]). **(C, D, E)** Alpha peak frequency distributions averaged over HC and TLE groups, and their comparison (computation procedure has been shown in Supplementary Materials). The color in **(E)** depicts the mean *Z* maps based on the ROI-wise comparison: blue indicates reduction of peak frequency. The color maps are thresholded at 5% FDR after permutation test. FDR, false discovery rate; HC, healthy control; PSDs, power spectral densities; ROI, region-of-interest; TLE, temporal lobe epilepsy. Color images are available online.

Patients with TLE showed significant deviations in spectral characteristics compared with HC (Fig. 1). Specifically, the patients with TLE showed reduced power in the alpha band and increased power in the low-frequency delta/theta bands (Fig. 1A). In addition, peak frequency within the alpha band was shifted to the left (slower) in TLE versus HC (Fig. 1B). In HC, distributions of the alpha peak frequencies over the brain exhibit a posterior-to-anterior pattern for HC (Fig. 1C), consistent with the observation of Mahjoory and colleagues (2020). This pattern is reversed in TLE (Fig. 1D). This difference is especially notable in the temporal and occipital regions.

### Information flow

**FIG. 2.**
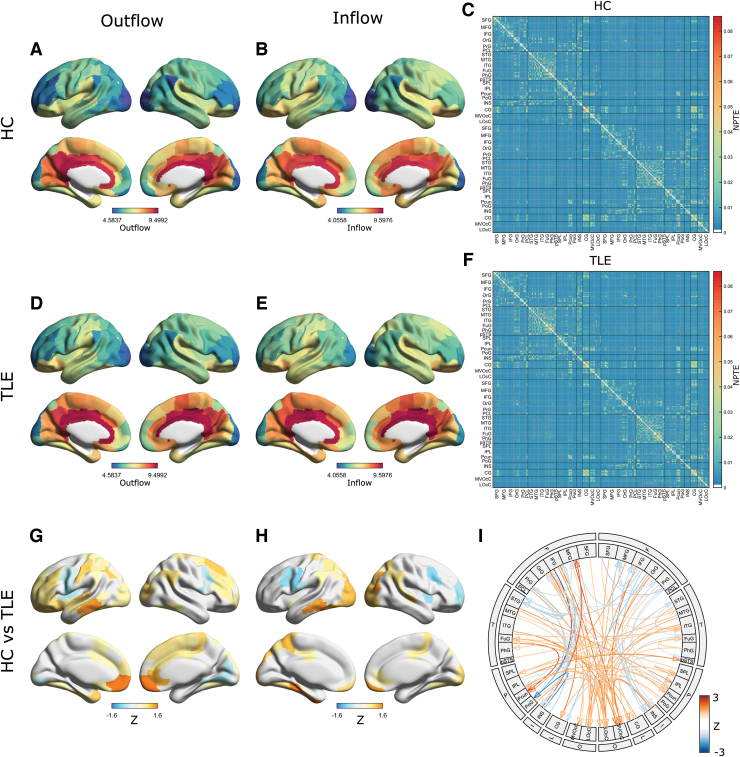
Information flow patterns in the alpha band based on NPTE. Regional information outflow and inflow patterns for HC **(A, B)** and TLE **(D, E)**, and average NPTE matrices for HC **(C)** and TLE **(F)**. For visualization, 1000 × values of regional NPTE are displayed. The color bar ranges are aligned between **(A** and **D)**, **(B** and **E)**, and **(C** and **F)**, respectively. The atlas module labels in the matrices are sorted left to right. (Bottom row) Alpha band disrupted information flow. Regional information outflow and inflow patterns **(G, H)** in TLE compared with HC. The color depicts the mean *Z* scores that survived in the ROI-wise comparison of regional NPTEs between groups using permutation tests, and colors in orange indicate increased information flow. The color maps are thresholded at 5% FDR. **(I)** Information flow connectogram in TLE compared with HC. The color depicts the average *Z* scores that are thresholded by *t*-tests with 0.1%-FDR correction. Only links with maximum absolute value of average *Z* scores between ROIs within each module were displayed. NPTE, normalized phase-transfer entropy. Color images are available online.

**FIG. 3.**
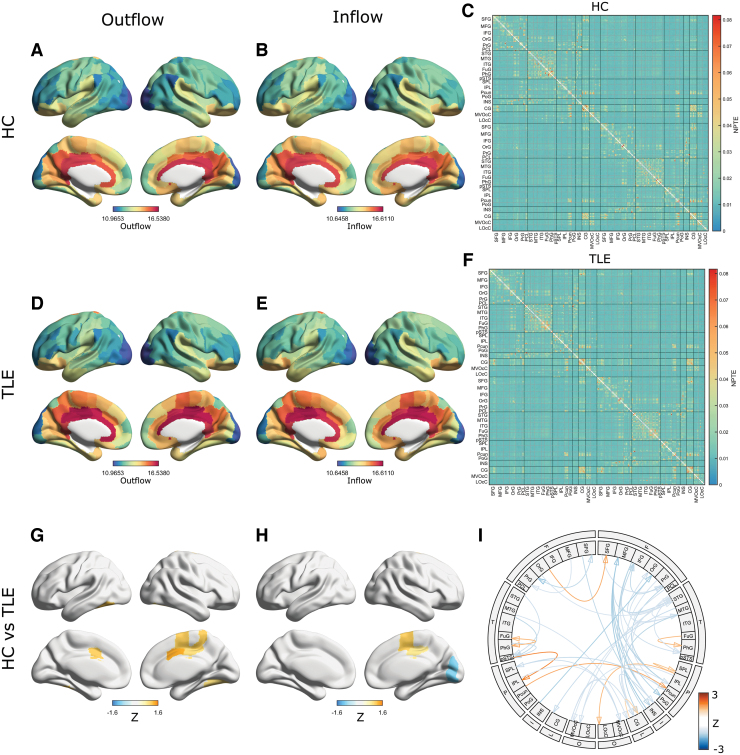
Information flow patterns in the delta/theta band based on NPTE. Regional information outflow and inflow patterns for HC **(A, B)** and TLE **(D, E)**, and average NPTE matrices for HC **(C)** and TLE **(F)**. For visualization, 1000× values of regional NPTE are displayed. (Bottom row) Delta/theta band disrupted information flow. Regional information outflow and inflow patterns **(G, H)** in TLE compared with HC. The color depicts the mean *Z* scores that survived in the ROI-wise comparison of regional NPTEs between groups using permutation tests, and colors in orange indicate increased information flow. The color maps are thresholded at 5% FDR. **(I)** Information flow connectogram in TLE compared with HC. The color depicts the average *Z* scores that are thresholded by *t*-tests with 0.1%-FDR correction. Only links with a maximum absolute value of average *Z* scores between ROIs within each module were displayed. Color images are available online.

Next, we examined the patterns of information flow in patients with TLE versus HC as represented by NPTE and dPTE. Information flow in specific frequency bands (i.e., alpha, 8–12 Hz; delta/theta, 1.5–7.5 Hz; and beta, 12–30 Hz) showed regional diversity in strength of outflow and inflow (Figs. 2 and 3 and Supplementary Fig. S2). For example, in HC, considering the alpha band, there was high information flow (i.e., with values larger than about 0.03) between regions with rich local connections at short distances (Fig. 2C) as defined in the Brainnetome atlas (Supplementary Fig. S1 in Supplementary Materials) (Fan et al., 2016). Patients with TLE showed similar regional diversity of information outflow and inflow as shown in Figure 2F. Such similar regional diversity was observed not only in the alpha band, but in both the lower (delta/theta) and in higher (beta) frequency bands (Fig. 3A–F and Supplementary Fig. S2A–F).

Although visually there seems to be little difference between the in- and outflow NPTE topographies and matrices for HC and TLE (e.g., Fig. 2A–C vs. D–F for the alpha band), there are clear quantitative differences in the patterns of information flow seen in patients with TLE when compared with patterns seen in HC (Fig. 2G–I). These differences were frequency band specific. Compared with HC, patients with TLE showed a relative increase in both outflow and inflow when considering alpha band activity (Fig. 2G–I). These differences were localized also: patients with TLE showed higher outflow from bilateral frontal and cingulate cortices, and higher inflow into bilateral occipital regions, compared with age-matched HC (Fig. 2G–I and Supplementary Fig. S7). In Supplementary Figures S8 and S9, we have shown group comparison of several components in the alpha-band NPTE matrix (Fig. 2C, F) and the alpha-band regional NPTE outflow (Fig. 2A, D) and inflow (Fig. 2B, E) to present intragroup variability of our data. We see, then, that the matrix components and regions showing large intragroup differences in terms of *Z* scores also show large intragroup differences in terms of raw metric values. In contrast to these differences seen in alpha band patterns, patients with TLE only showed a moderate degree of change in beta band information flow when compared with HC (Supplementary Fig. S2G–I), and only a limited change of information flow within the delta/theta band (Fig. 3G–I) compared with HC. For example, TLE patients showed some increased delta/theta outflow from limited bilateral frontal regions and reduced delta/theta outflow and inflow within several occipitoparietal regions. Collectively, these findings indicate that regional information outflow and inflow are significantly altered in patients with TLE and that these changes vary across different frequency bands and are seen most prominently in the alpha band.

### Directional information flow

**FIG. 4.**
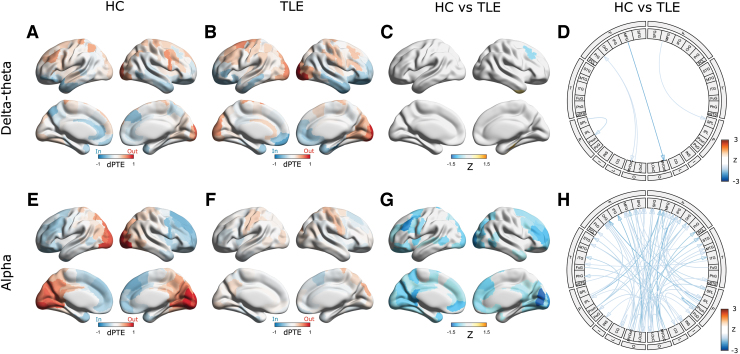
Regional directionality of information flow. Average regional dPTEs for HC and TLE for the delta/theta **(A, B)** and alpha **(E, F)** bands. Red indicates outflow directionality and blue indicates inflow directionality. Disrupted directionality patterns in TLE are represented as the *Z*-score maps for the delta/theta **(C)** and alpha **(G)** bands. The color depicts the mean *Z* scores that survived in the ROI-wise comparison of regional dPTEs between groups using permutation tests with 5%-FDR correction. Blue (orange) denotes decreased (with respect to increased) directionality. **(D, H)**: Disrupted information directionality in TLE versus HC for the delta/theta **(D)** and alpha **(H)** bands, depicted as connectograms. The color depicts the average *Z* scores thresholded by *t*-tests with 5%-FDR correction for the delta/theta **(D)** band and 1%-FDR correction for the alpha **(H)** band, respectively. For visualization, only a link with maximum absolute value of average *Z* scores between ROIs within each module is displayed. dPTE, directional phase-transfer entropy. Color images are available online.

Next, we examined whether TLE is associated with altered directionality of information flow in the brain. To this end, we examined the regional patterns of dPTE in patients with TLE compared with HC. Consistent with previous reports (Hillebrand et al., 2016), we found distinct patterns of directional information flow within the alpha, delta/theta, and beta oscillatory bands in our HC. For example, in the alpha and beta bands, HC showed a predominant occipital outflow with an overall pattern of posterior-to-anterior information flow (Fig. 4E and Supplementary Fig. S4A); this was frequency-specific and not apparent in the delta/theta band. Compared with this pattern in HC, the patients with TLE showed significant reductions in directional information flow in the alpha (Fig. 4F–H) and beta (Supplementary Fig. S4B–D) bands. The connectogram for the dPTE matrices indicates that the reduction of alpha band information flow directionality mostly involves connections from the occipital cortex to the frontal regions. This reduction was also frequency specific; when the delta/theta band was considered, the patients with TLE only showed minimal differences of dPTE within the delta/theta band compared with HC (Fig. 4C, D). To show intragroup variability, group comparison of several components in the alpha band dPTE matrix (Supplementary Fig. S3B, E) and the alpha band regional dPTE (Fig. 4E, F) is shown in Supplementary Figure S10. The values of the matrix components and regions in TLE are significantly less than those in HC, reflecting significant reductions in directionality for the TLE group, as shown in Figure 4G and H.

In Supplementary Figure S6, group comparisons of mean global strength of dPTE in the delta/theta, alpha, and beta bands are shown. This scalar measure was derived by taking an average of absolute values of dPTE-matrix (i.e., |dPTE|) across all the matrix components since dPTE has both positive and negative values. For the alpha and beta bands, this measure was significantly different between the groups (Supplementary Fig. S6B, C), but not so for the delta band (Supplementary Fig. S6A). The significant differences for the alpha and beta bands may reflect significant reductions in edge- and region-level directionality in the TLE group for the alpha and beta bands (Fig. 4G, H, and Supplementary Fig. S4C, D). An estimation of area under the curve for an receiver operating characteristic analysis examining the omnibus alpha and beta band measure ability to distinguish between TLE and HC resulted in values larger than 0.80 (Supplementary Fig. S6D. See also Supplementary Fig. S13 for comparison of dPTE with relative power and Imcoh). This indicates that such omnibus measures have a potential to serve as biomarkers for TLE/HC discrimination.

### Correlations with spike frequency and epilepsy duration

**FIG. 5.**
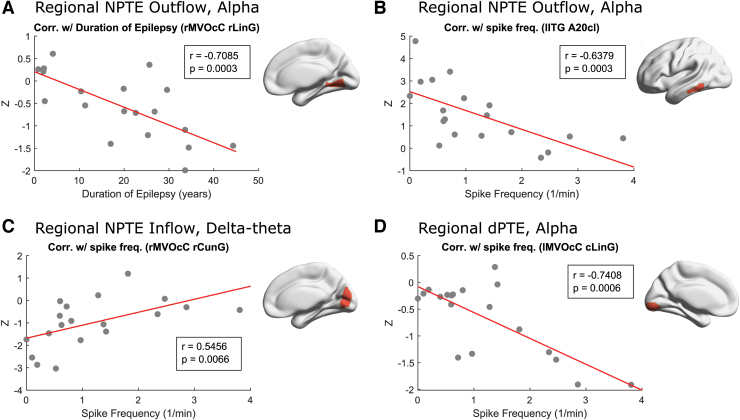
Correlations between regional information flow and spike frequency and with epilepsy duration in TLE patients. **(A, B)** Regional information outflow at right rostral lingual gyrus (“rMVOcC rLinG”) and left inferior temporal gyrus [caudolateral of area 20] (“lITG A20cl”) was negatively correlated with the spike frequency in the alpha band. **(C)** Regional information inflow at right rostral cuneus gyrus (“rMVOcC rCunG”) is positively correlated with the spike frequency in the delta/theta band. **(D)** Regional information directionality at left caudal lingual gyrus (“lMVOcC cLinG”) is negatively correlated with the duration of epilepsy in the alpha band. The definition of brain regions and their abbreviations are shown in Supplementary Tables S1 and S2. Color images are available online.

Next, we investigated the associations between indices of information flow in patients with TLE and clinical variables reflecting their disease burden. Specifically, we examined the correlations between NPTE and dPTE within specific frequency bands with the duration of epilepsy and with the frequency of spikes recorded during the clinical MEG scan in our TLE patient cohort. We found several associations: first, reduced information outflow at the right rostral lingual gyrus within the alpha band was significantly associated with a longer duration of epilepsy (Fig. 5A, *r* = 0.7085, *p* = 0.003). Second, increased spike frequency showed significant associations with several changes in regional information flow, reduced information outflow at the left inferior temporal gyrus within the alpha band (Fig. 5B), increased information inflow at right rostral cuneus gyrus within the delta/theta band, and reduced directionality of information flow at the left caudal lingual gyrus within the alpha band (Fig. 5C, D).

## Discussion

In this study, we used MEGI to record spontaneous electromagnetic brain activity during rest in patients with medically refractory TLE and with age-matched HCs. Following source localization of this resting-state MEG data, we computed PTE to examine information flow (NPTE) and relative directional information flow (dPTE) between brain regions. Both NPTE and dPTE are pairwise measures between brain regions, for example, *R*_1_ and *R*_2_; while NPTE measures the strength of information flow from *R*_1_ to *R*_2_ and from *R*_2_ to *R*_1_, dPTE measures the relative directionality between these paired measures. We discovered frequency-specific features of abnormal information flow in TLE patients, some of which were associated with clinical features of their disease.

### Disruption of information flow

Collectively, we saw that both indices—NPTE and dPTE—were altered in patients with TLE compared with HC. This indicates that both dimensions of information flow, the strength and direction, are altered in TLE. Moreover, this alteration appears to be frequency specific as well as regionally dependent. Directionality of information flow was significantly reduced in patients with TLE, and primarily in the alpha and to a lesser extent in the beta band. This observation contrasts with a similar study of information flow in patients with Alzheimer's disease (AD) (Engels et al., 2017), where the reduction of the directionality of information flow was most prominent in the beta band in comparison with other bands. This may be accounted for by the differences in spectral content of the resting-state background neuronal activities in AD and TLE cohorts.

In this study, we also examined the local outflow and inflow of information as well as directionality by considering regional NPTE. Regional NPTE patterns indicate that the reduction of information directionality observed in regional dPTE results from an increase in both information outflow and inflow in patients with TLE versus in HC. Especially in the frontal and occipital regions, these local increases in outflow and inflow cancel each other out, and the result is a net reduction in directionality of information flow in TLE.

Theoretically, PTE and signal power can be independent measures computed on a time series since the PTE measure is based on phase timecourse, in which signal amplitude is omitted, that is, the PTE should be independent of signal power. Nevertheless, we empirically investigated if regional spectral power was associated with regional dPTE (the results are described in section 6.4 of the Supplementary Materials). To our surprise, in the alpha and beta bands, but not delta, we found that the correlation between regional relative power and regional dPTE also showed a clear posterior-to-anterior pattern (Fig. 4), a pattern also observed in the regional spectral power (Supplementary Figs. S11 and S12). These results suggest a complex relationship between spectral power and PTE measures.

### Associations of information transfer with clinical measures

We examined the correlation of regional NPTE and dPTE in patients with TLE with several clinical measures of disease severity: spike frequency and duration of epilepsy. Information transfer in several brain regions was correlated with these clinical measures; these correlations were observed mainly in the alpha band and to a lesser extent in the delta/theta band, but not in the beta band. Specifically, alterations in information flow in the right posteromedial temporal cortex show a relationship with duration of epilepsy; and alterations in information flow in the left inferior-lateral temporal cortex and in medial occipital cortex show a relationship with spike frequency.

We performed the same analysis using regional ImCoh (Supplementary Fig. S5A–C) and also examined the associations of ImCoh with clinical characteristics. We, however, did not find significant correlations for regional ImCoh in any frequency bands. In addition, there was not any significant correlation between global mean ImCoh and clinical measures. This is partly consistent with the result described in an earlier published work from our laboratory (Englot et al., 2015) on the correlation between global mean ImCoh and duration of epilepsy in patients with TLE. This suggests that PTE metrics may outperform ImCoh in detecting the features of neuronal activity more relevant to clinical measures in patients with TLE. This improved performance may come from the fact that, unlike ImCoh, the TE metric can capture both directionality and strength of network connections.

Interictal spike frequency has proven to be of limited value in isolation as a predictor of seizure onset zone (Ngo et al., 2017), and for severity of epilepsy (Selvitelli et al., 2010). However, the association of frequent spikes with the marker that we here identify—disruption in information flow—offers us a potential mechanistic explanation that links interictal spikes to end effects. For example, spikes interrupt neuronal activity, as reflected in changes in PTE; changes in PTE may allow us to understand patterns of cognitive change in focal epilepsy. This may lead to an improved understanding of the mechanism underlying the association between interictal spikes and cognitive deficits (Faught et al., 2018). Likewise, changes in regional connectivity may provide an intermediary by which to better quantify epilepsy severity (beyond duration/years since diagnosis) for the purpose of understanding specific deficits.

### Limitations of this study

We observed overall slowing of the background frequencies and a decrease in peak frequency within the alpha band. This was unlikely to be an effect solely of state, as all epochs were selected from waking record. However, people with epilepsy were more likely to have been sleep deprived before their clinical study and thus may have been drowsier as a group. In addition, they were on antiseizure medications, which may have had sedating effects and led them to have been drowsier, accounting for a leftward shift in alpha peak and a change in topology.

Our analysis of PTE is, as a method, also applicable to resting-state signals with higher frequency bands such as the low gamma band (30–55 Hz). In an exploratory analysis, we computed low gamma band PTE for our data. However, we found that computed PTE values for several pairs of brain regions were negative, which is theoretically impermissible given bias correction. This is probably due to the low signal-to-noise ratio of low gamma band signals. One way to correct this calculation error may be to consider using a longer timecourse so that the joint probability density for evaluation of PTE can be determined more precisely.

In the current study, we examined the data in specific frequency bands and prespecified brain regions of interest to investigate frequency-specific abnormality of information flow in TLE. An alternative approach would have been to use a data-driven technique for extracting relevant frequency bands and ROIs for such investigations, such as multiband independent component analysis (Nugent et al., 2017) and dynamic mode decomposition (Brunton et al., 2016). Future studies are needed to compare these different approaches in terms of reliability and sensitivity in revealing altered network dynamics in TLE.

## Conclusions

This study investigates changes in information flow in people with TLE by applying phase-transfer entropy measures, NPTE and dPTE, to resting-state magnetoencephalographic data. Deficits of information flow are seen in TLE most prominently in the alpha band. While HCs exhibit a clear posterior-to-anterior directionality of information flow, as measured using dPTE in patients with TLE, this pattern is significantly altered in the frontal and occipital regions. This change in pattern of directionality is mainly due to an alteration of counterbalance between activated regional information outflow and inflow, as measured using NPTE. The changes in information flow in the alpha band in selected brain regions are correlated with several clinical measures related to epilepsy, including interictal spike frequency and disease duration. Analyzing MEGI using both dimensions of information flow strength and direction provides a powerful approach to investigate network disruption in TLE.

## Supplementary Material

Supplemental data
