# Supplementary material for: Magnetoencephalography Imaging Reveals Abnormal Information Flow in Temporal Lobe Epilepsy
Source: Brain Connect. 2022 May 12;12(4):362–73. doi: 10.1089/brain.2020.0989 (PMC9131359; doi:10.1089/brain.2020.0989)
Supplement: Supplemental data [file Supp_Data.pdf]

Supplemental Materials:
MEG Imaging reveals abnormal information flow in TLE

Kiwamu Kudo, Hirofumi Morise, Kamalini G. Ranasinghe, Danielle Mizuiri, Abhishek S. Bhutada, Jessie Chen, Anne Findlay, Heidi E. Kirsch, and Srikantan S. Nagarajan

1. Peak frequency distribution around the alpha band

Distributions of peak frequencies around the alpha band (8–12 Hz) for each participant was detected, and their means obtained by averaging over each group were shown in Figures 1 D–E in the main text. We employed the method described in Mahjoory et al. (2020) for detecting the peak frequencies: first we estimated aperiodic component of PSD such as $1/f$ noise between 1 and 45 Hz using the FOOOF algorithm (Haller et al., 2018), and then subtracted it from the original PSD. After that, we applied the MATLAB 'findpeaks' function to the corrected PSD and extracted the frequency with maximum PSD amplitude. For several participants not having peak frequency within the range of 8–12 Hz on several ROIs, a peak frequency with the strongest power or a frequency with maximum power in the range of 6–14 Hz was defined as a peak frequency.

2. Distances between brain regions based on Brainnetome Atlas

In the main text, we mentioned distances between brain regions in the context of showing the feature of the PTE matrices (e.g., Figs. 2C and F). Here we show how we estimated the distances between ROIs based on the Brainnetome atlas dataset.

The binary data of structural connections between brain regions for Brainnetome Atlas (Fan et al., 2016) are provided as a csv file at the Brainnetome web page. It also provides MNI coordinates for each ROIs as an xlsx file [1]. Based on these data, we estimated shortest-path length between brain regions. First we calculated euclidean distances between directly connected ROIs using their MNI coordinate values and obtained a distance-weighted

[1] Tables 1 and 2 are based on the xlsx file.

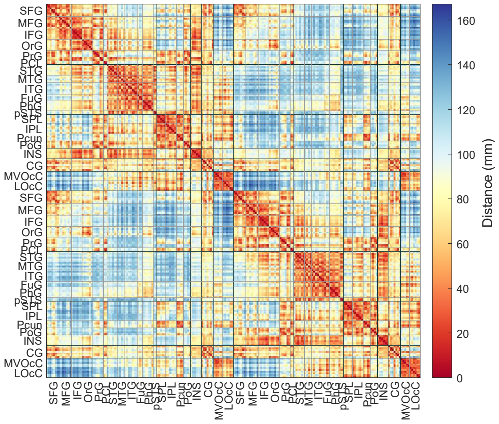

Figure S1: Distances between brain regions evaluated based on Brainnetome Atlas database.

matrix. After that, for all the pairs of ROIs, the length of the shortest possible path between the ROIs was computed by Floyd-Warshall algorithm that is implemented in a MATLAB toolbox, Brain Connectivity Toolbox (Rubinov and Sporns, 2010).

Figure S1 shows the the obtained shortest-path lengths. By comparing the shortest-path-length matrix (Fig. S1) with the PTE matrices (e.g., Figs. 2C and F), we can find that large information flow shown in the main text appears between brain regions with short distance less than approximately 8 cm (having warm color).

3. Normalized phase transfer entropy (NPTE) in the beta band

Computation results of PTE for beta band are shown in Figure S2. In beta band, information outflow increases in the superior frontal gyrus and orbital gyrus, and it decreases in the cuneus and occipital gyri (Fig. S2G). On the other hand, information inflow decreases in the middle and inferior frontal gyri (Fig. S2H).

4. Directional phase transfer entropy (dPTE)

In the main text, we have focused on the results on regional dPTE for the delta-theta and alpha bands. Here we show dPTE matrices for all the

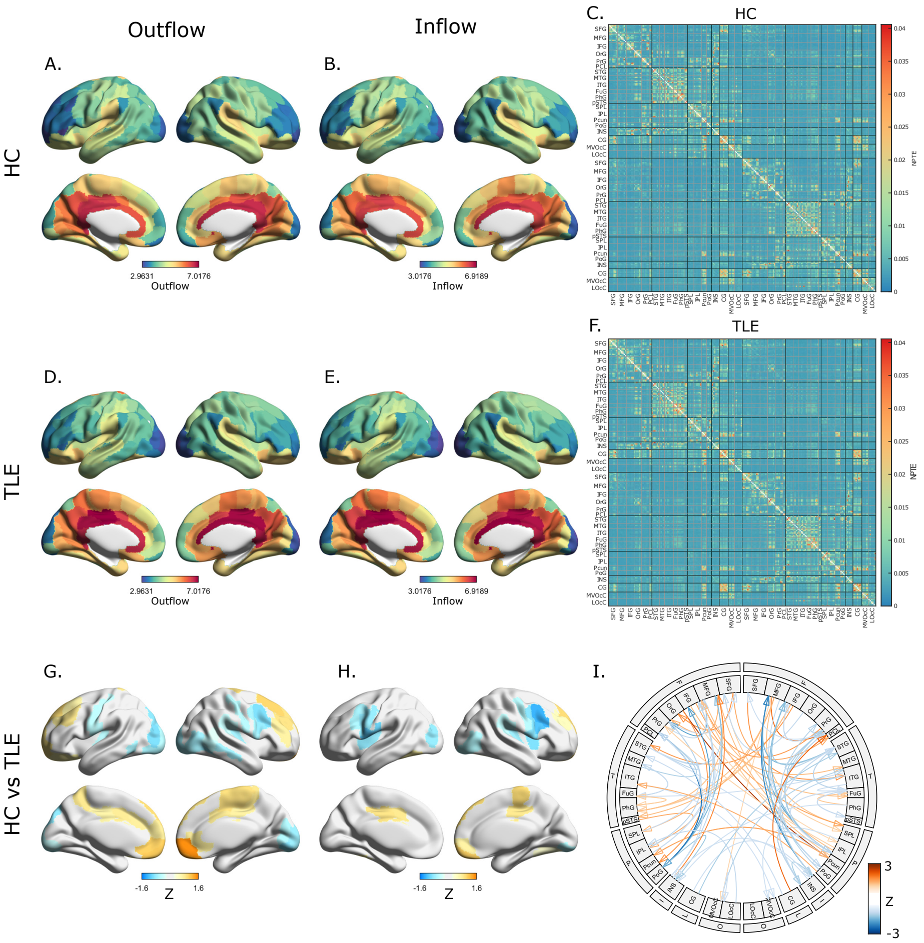

Figure S2: Information flow patterns in the beta band based on normalized phase transfer entropy (NPTE). Regional information out- and in-flow patterns for HC (A,B) and TLE (D,E), and average NPTE matrices for HC (C) and TLE (F). (*Bottom row*) Beta-band disrupted information flow. Regional information out- and in-flow patterns (G and H) in TLE compared with HC. The color depicts the mean Z scores that survived in the ROI-wise comparison of regional NPTEs between groups using permutation tests, and colors in orange indicate increased information flow. The colormaps are thresholded at 5% FDR. *Panel I*: Information flow connectogram in TLE compared with HC. The color depicts the average Z scores which are thresholded by t-tests with 0.1%-FDR correction. Only links with maximum absolute value of average Z scores between ROIs within each module were displayed.

frequency bands and regional dPTE in the beta band.

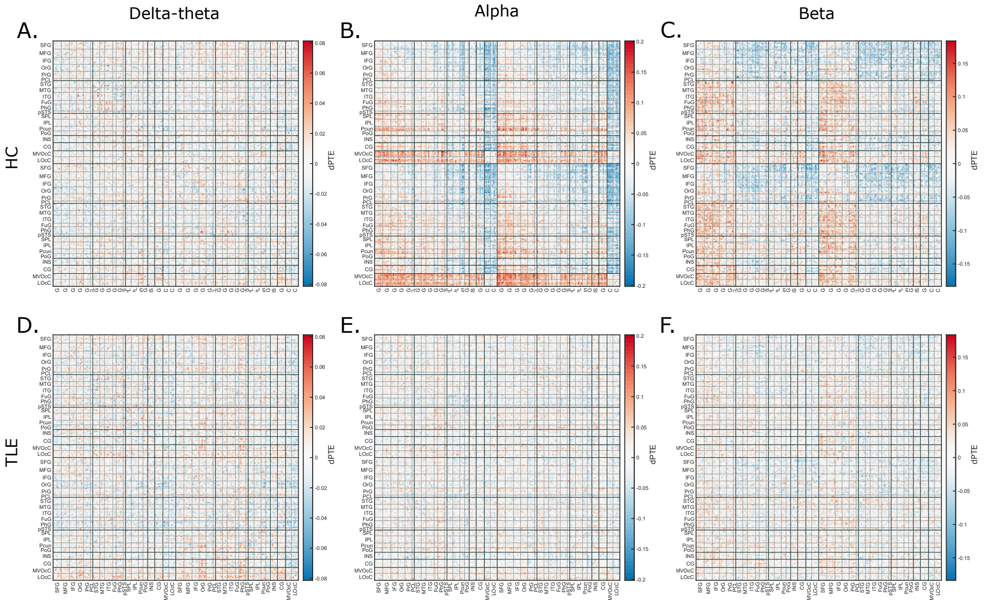

Figure S3: Average dPTE matrices for HC and TLE in the delta-theta (A,D), alpha (B,E), and beta (C,F) bands. For comparisons between HC and TLE, the colorbar ranges are aligned between (A) and (D), (B) and (E), and (C) and (F), respectively.

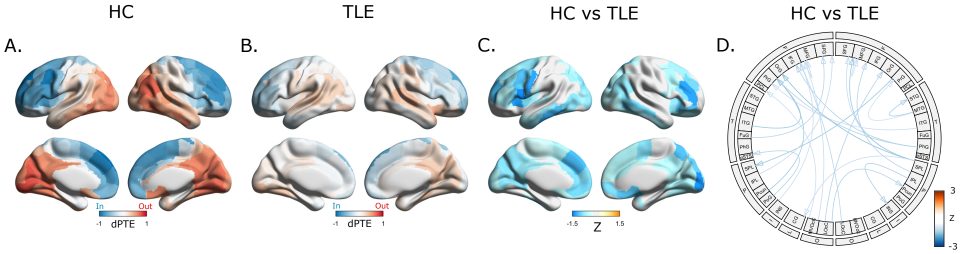

Figure S4: Beta-band regional directionality of information flow for HC and TLE (A and B). Red (blue) indicates outflow (resp. inflow) directionality. (C) Disrupted directionality patterns in TLE as the Z-score map. The color depicts the average Z scores that survived in the ROI-wise comparison of regional dPTEs between groups using permutation tests with 5%-FDR correction. Blue (orange) denote decreased (resp. increased) directionality. (D) Disrupted information directionality depicted as connectograms. The color depicts the average Z scores thresholded by t-tests with 1%-FDR correction. Only links with maximum absolute value of average Z scores between ROIs within each module were displayed.

The dPTE matrices for delta-theta, alpha, and beta bands are shown in Figure S3. It should be noting that the dPTE matrix is an anti-symmetric

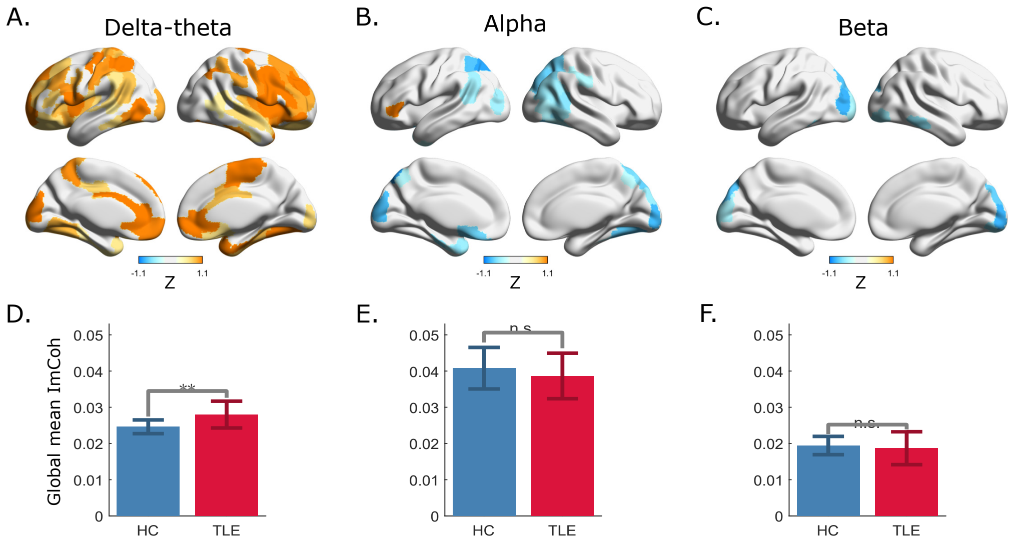

Figure S5: (A,B,C) Neural synchrony evaluated using ImCoh in TLE compared with age-matched HC. The color depicts the mean Z-score maps from the ROI-wise comparison of global imaginary coherence between groups in the delta-theta, alpha, and beta bands. Blue indicates hyposynchrony, and orange indicates hypersynchrony. The color maps are thresholded at 5% FDR (n = 19, TLE; n = 20, age-matched HC) after permutation tests. (D,E,F) Mean global synchrony estimated by using ImCoh averaged across the 210 Brainnetome cortical regions for the delta-theta, alpha, and beta bands. Significant hypersynchrony was observed in TLE in delta-theta band ($^{**}p < 0.01$, t-tests). The data are shown as mean ± SD (arbitrary units).

one. The regional dPTE in the beta band for HC and TLE, and their comparisons are shown in Figure S4.

5. Neural Synchrony

Neural synchrony between ROI-level brain areas was evaluated by computing the imaginary part of the coherence 'ImCoh'. The coherence for a pair of source timecourses, x and y, within a frequency band B was defined by

$$\mathrm{Coh}_{xy} = \frac{\sum_{k=1}^{K} \sum_{f \in B} X_k(f) Y_k^*(f)}{\sqrt{\sum_{k=1}^{K} \sum_{f \in B} |X_k(f)|^2 \sum_{k=1}^{K} \sum_{f \in B} |Y_k(f)|^2}}, \tag{S1}$$

where $X_k(f)$ and $Y_k(f)$ are the k-th windowed Fourier transforms of x and y, respectively (Nolte et al., 2004). K is the total number of windows. We

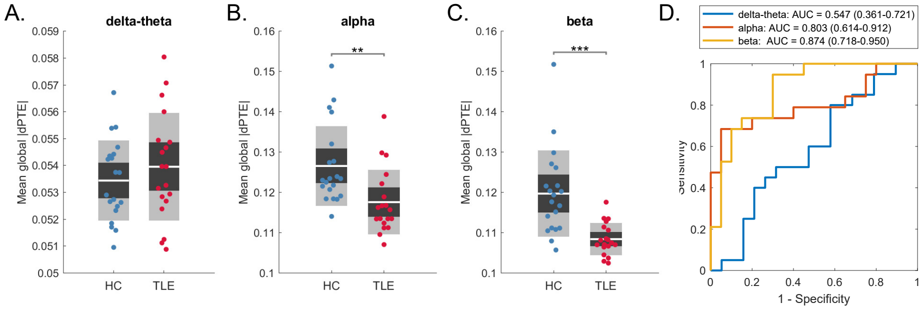

Figure S6: (A,B,C) Group comparisons of mean global strength of dPTE in the delta-theta, alpha, and beta bands. Significant difference was observed in the alpha band (**$p = 0.0036 < 0.01$ in permutation test) and beta band (***$p = 1.8^{-4} < 0.001$ in permutation test). There was no significant difference in the delta-theta band. (D) ROC curves of the global strength of dPTE for the delta-theta, alpha, and beta bands.

used Hanning window as a window function, the length of which was 3.41 sec ($2048/f_s$). ImCoh is the imaginary part of Coh_{xy}, and we defined it as $\text{ImCoh}_{xy} = |\text{Im Coh}_{xy}|$. The connectivity strength at each ROI, regional ImCoh, was estimated by averaging across all Fisher's Z-transformed ImCohs (Guggisberg et al., 2008), i.e., using $\tanh[\frac{1}{N_{\text{ROI}}-1}\sum_y \tanh^{-1} \text{ImCoh}_{xy}]$.

In patients with TLE, neuronal synchrony within the alpha and beta bands was reduced (hyposynchrony) compared to controls, whereas neuronal synchrony within the delta-theta band was increased (hypersynchrony) compared to controls. Anatomically, alpha hyposynchrony was found especially in posterior parietal and occipital regions (Fig. S5B). In contrast, delta-theta hypersynchrony was found especially in frontal regions (Fig. S5A). Interestingly, these distribution patterns of hypo- and hyper-synchrony are quite similar to those observed in MEGI in AD patients (Ranasinghe et al., 2020).

6. Supplementary results for NPTE and dPTE

6.1. Mean global strength of PTE directionality

Like ImCoh, a mean global measure averaged over all the ROI pairs (i.e., edges) can be defined for the directionality of phase transfer entropy (dPTE). Since dPTE takes both positive and negative value, it is appropriate to define such a scalar measure by taking an average of absolute values of dPTE-matrix components (i.e., |dPTE|). This quantifies global strength of PTE directionality. Here we show group comparisons of the strength between HC

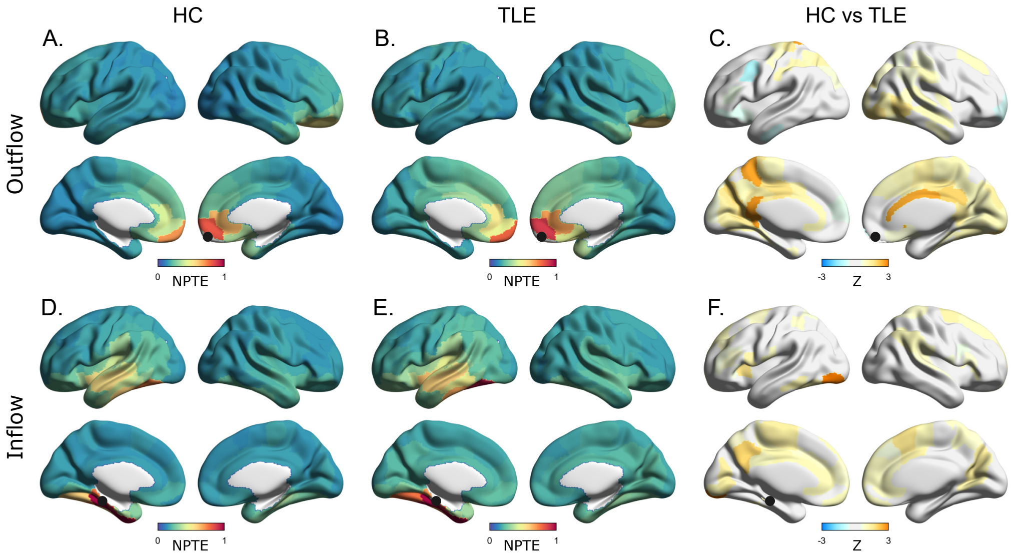

Figure S7: Group comparison of alpha-band seed-based PTE out- and in-flow between HC and TLE. Seeds for PTE outflow and inflow were chosen based on maximal group differences in Z-scores between HC and TLE across the entire matrix. For PTE outflow, the seed region was the orbital gyrus [medial area 11]. For PTE inflow, the seed region was the left lateral posterior parahippocampal gyrus. (see Figs. 2G and H in the main text). The seed ROIs were denoted as black filled circles. For the NPTE rendering (A, B, D, and E), a converted quantity, $\tanh[\mathrm{NPTE(i)}/(\mathrm{NPTE}_{\max}/3)]$, was used for visualization, where $\mathrm{NPTE}_{\max} = \max_{j=1,...210} \mathrm{NPTE(j)}$. (C, F): Information flow from and into the seed regions in patients with TLE were larger than those in HCs. The color maps which represent Z scores are thresholded at 5% FDR after permutation tests. Significant statistical difference was observed between the groups. The large difference in the regional PTE outflow at the right orbital gyrus [medial area 11] mainly originates in the information flow into right cingulate gyrus [rostroventral area 24], left precuneus [medial area 5], and left cingulate gyrus [ventral area 23] (mean Z-scores in these regions are larger than 2). The large difference in the regional PTE inflow at the lateral posterior parahippocampal gyrus mainly originates in the information flow from left inferior occipital gyrus (mean Z-scores in this region is larger than 2).

and TLE for the delta-theta, alpha, and beta bands. The results were shown as box plots. A Matlab tool 'notBoxPlot' (Campbell, 2016) was used for making the box plots.

There was no significant difference between HC and TLE in the delta-theta-band global directionality strength (Fig. S6A). On the other hand, significant reductions in the directionality strength in TLE were observed for the alpha and beta bands (Figs. S6B and C). It is worth noting that these reduc-

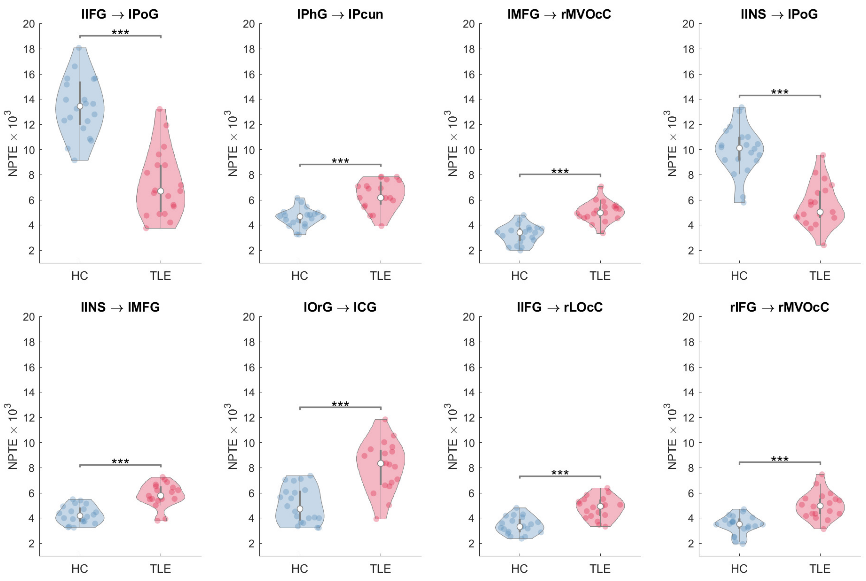

Figure S8: Group comparison of several components in the alpha-band NPTE matrix (Figs. 2C and F) between HC and TLE. The comparisons at the ROI pairs (i.e., edges) that have large mean absolute Z scores (> 2; see Fig. 2I in the main text) were depicted. Clear discrimination between the groups with $p < 0.001$ in the permutation test for NPTE values was observed at all the selected ROI pairs. The panels are arranged from the upper left in descending order of absolute value of Z score.

tions do not represents information-flow reduction in TLE. As we mentioned in the main text in the context of comparing the results of alpha-band dPTE with those of NPTE (e.g., Figs. 4G-H and Figs. 2G-I), the directionality-strength reductions rather originate in cancellation between the larger information out- and in-flow in TLE compared with HC (see Figs. 2G-I).

To check performance of mean global measures for TLE/HC discrimination, Receiver Operating Characteristic (ROC) curve analysis was also performed (Fig. S6D). Logistic, non-parametric method (Qin and Hotilovac, 2008) was used for their Area Under Curve (AUC) estimates. The alpha- and beta-band mean global |dPTE| have a potential to become a good scalar diagnostic measure for TLE in the sense that AUC is greater than 0.80 (Fig. S6D).

6.2. Seed-based PTE

In the main text, we investigated regional information outflow and inflow based on PTE matrix. Such an investigation is effective and convenient for

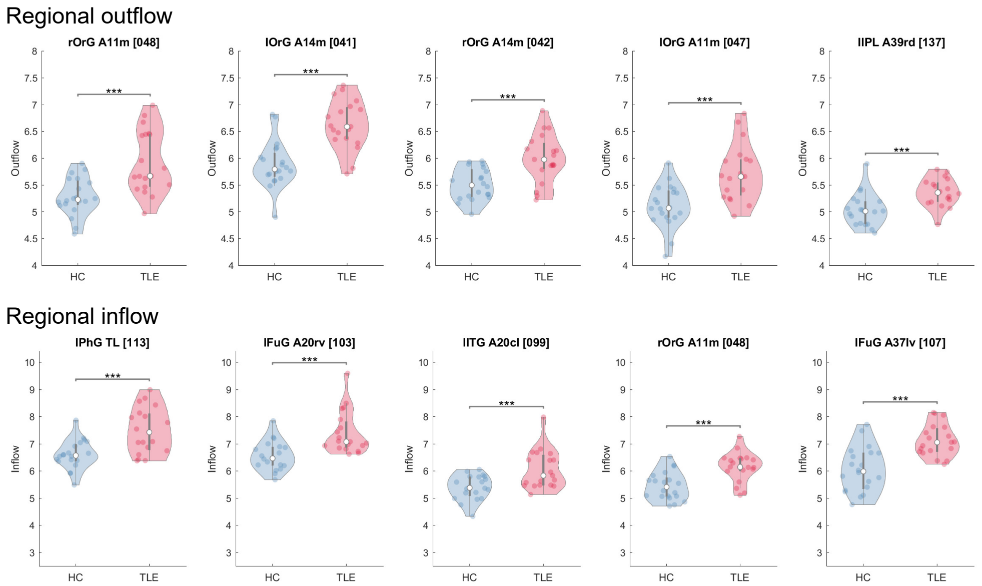

Figure S9: Group comparison of alpha-band regional NPTE outflow (Figs. 2A and D) and inflow (Figs. 2B and E) between HC and TLE. Clear discrimination between the groups ($p < 0.001$ in the permutation test) were observed at 7 and 9 ROIs for regional outflow and inflow, respectively. The comparisons at 5 ROIs among them that have top 5 absolute values of Z score (Figs. 2G or H) were depicted, being arranged from the left in descending order of the values.

data-dimension reduction and regional comparisons. There is however yet another approach to explore regional features: considering seed-based connectivity. Detailed consideration of seed-based connectivity is out of scope in this paper, but here we exemplify seed-based PTEs and their comparisons between HC and TLE; two ROIs where the greatest inflow and outflow difference within the alpha band were observed (see Figs 2G and H in the main text) were picked up as seeds.

Figure S7 shows the results. Information flow from and into the seed regions in patients with TLE were larger than those in HCs (Figs. S7C and F), reflecting aggregate increases in the outflow and inflow at the seed regions in TLE (Figs. 2G and H). This seed-based analysis further clarifies that the aggregate increases emerged in a region-specific way; several regions mainly contribute to the increases. This kind of seed-based approach, especially by putting a seed on ROI which highly correlates with clinical measures, such as spike frequency and duration of epilepsy, might be useful to investigate

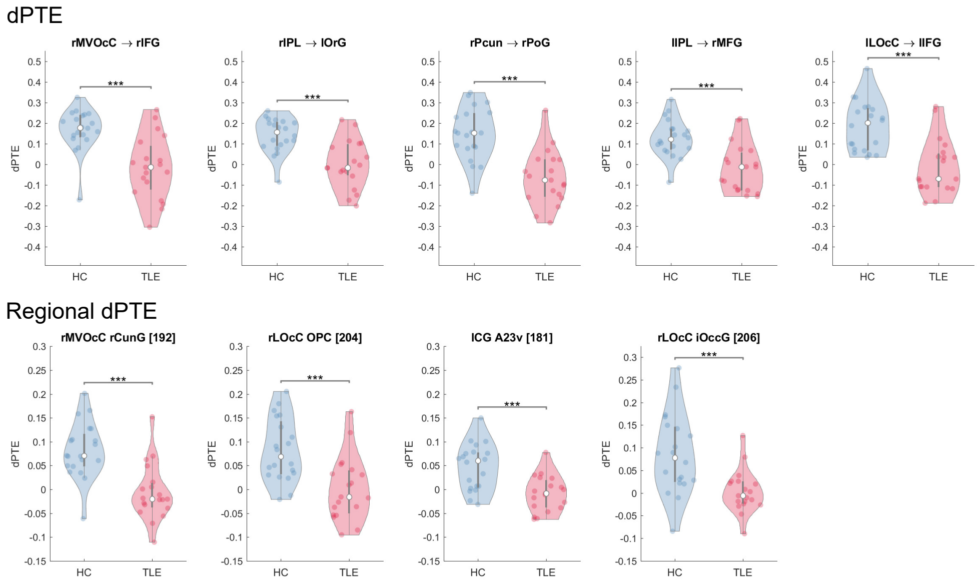

Figure S10: (*Upper panels*:) Group comparison of several components in the alpha-band dPTE matrix (Figs. S3B and E) between HC and TLE. The comparisons at the ROI pairs (i.e., edges) that have large mean absolute Z scores (> 1.6; see Fig. 4H in the main text) were depicted. Clear discrimination between the groups with $^{***}p < 0.001$ in the permutation test for dPTE values was observed at all the selected ROI pairs. The panels are arranged from the left in descending order of absolute value of Z score. (*Lower panels*:) Group comparison of alpha-band regional dPTE (Figs. 4E and F). The comparisons at the ROIs providing provide clear discrimination between the groups ($^{***}p < 0.001$ in the permutation test) were depicted. The panels are arranged from the left in descending order of the absolute value of Z score (Fig. 4G).

information-network contribution to the clinical measures, but this is a future study.

6.3. Group comparisons of NPTE matrix, regional NPTE and dPTE

In the main text, we discussed the deviation of connectivity in people with TLE versus HCs by introducing the Z score based on the connectivity in the HC group. This treatment makes a little hard to see intra-group variability for the connectivity measures, especially for those in the HC group. In order to clear the intra-group variability, we here exemplify group comparisons of the measures for several ROIs or edges that show clear group differences, focusing on the alpha band. The variability was visually shown by violin plots using a Matlab tool for them (Bechtold, 2016).

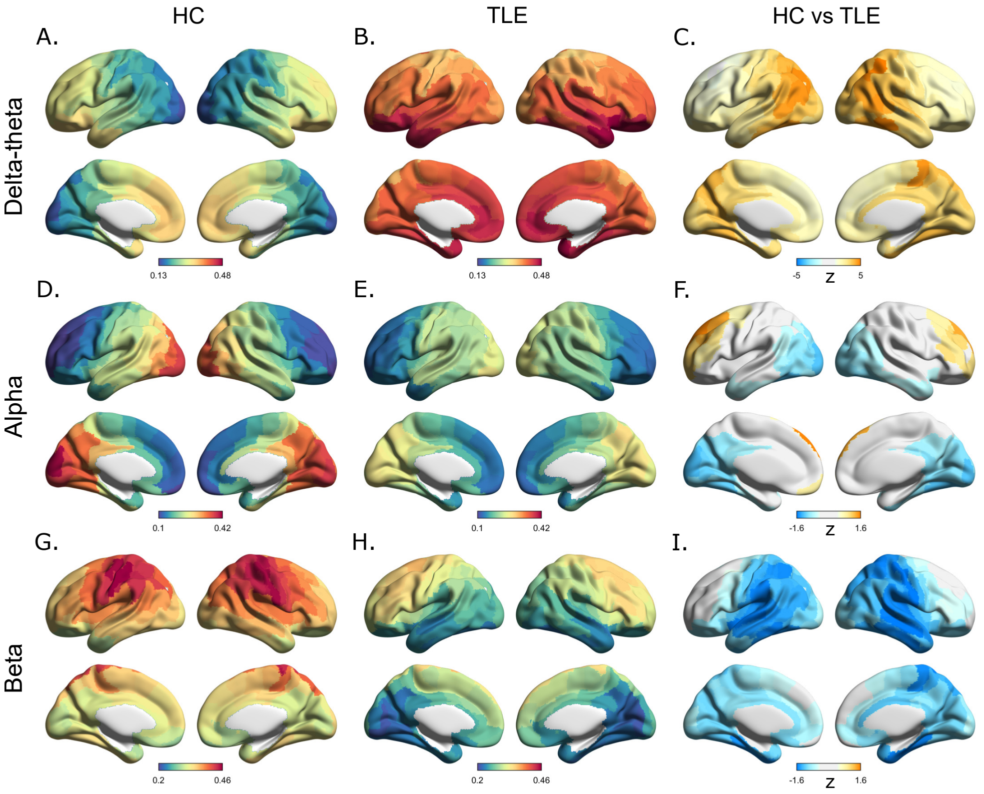

Figure S11: Relative-power spatial patterns for HC, TLE, and their difference represented as Z-score map for the delta-theta (A, B, and C), alpha (D, E, and F), and beta (G, H, and I) bands. The Z-score maps denote change in the relative-power pattern in TLE compared with HC-average pattern. The color in the Z-score maps represents mean Z scores thresholded by 5% FDR after permutation tests. Blue (orange) denote negative (resp. positive) change in relative power.

Figures S8 show group comparison of several components in the alpha-band NPTE matrix (see Figs. 2C and F). By comparing these figures with the connectogram (Fig. 2I), it is identified that a mean NPTE value for HC is larger (smaller) than that for TLE when mean Z is positive (resp. negative), which is along with the definition of Z score.

6.4. Correlation between spatial patterns of dPTE and those of relative power

As we showed in the main text, the dPTE for HC had a clear posterior-to-anterior pattern in the alpha band (see Fig. 4E). The back-to-front pattern is visually like a well-known mean relative-power spatial pattern [see, e.g.,

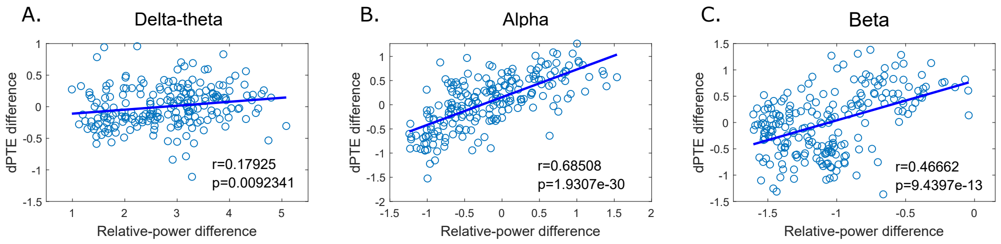

Figure S12: Similarity between group differences in regional dPTE values and those in the delta-theta (A), alpha (B), and beta (C) relative power. Z scores standardized by HC values [a standard definition was used here: $Z = (w - w_{\mathrm{HC}})/\sigma_{\mathrm{HC}}$, in which the multiplication of $\mathrm{sgn}(w_{\mathrm{HC}})$ is not included] were computed for each ROI, and then the group differences were quantified by averaging the Z scores over TLE group. The number of circular dots is accordingly equal to that of ROIs, i.e., 210. The similarity was evaluated by computing Pearson's correlation coefficient. A strong positive correlation ($r \sim 0.7$) was observed in the alpha band.

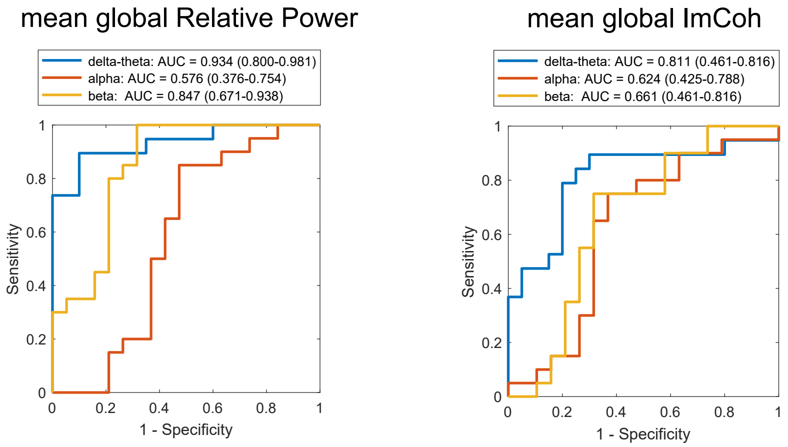

Figure S13: ROC curves of the global strength of relative power and ImCoh for the delta-theta, alpha, and beta bands.

Fig. 4 in Hillebrand et al. (2012)], and our HC data also provide such a back-to-front pattern in the alpha band (Fig. S11D). Theoretically, regional dPTE and relative power can be independent measures computed on a time-series since PTE measure is based on phase timecourse, in which signal amplitude is omitted. Nevertheless, we empirically investigated if regional spectral power was associated with regional dPTE. To our surprise, in the alpha and beta bands, but not delta, we found that the correlation between regional relative power and dPTE also showed a clear posterior-to-anterior pattern, a

pattern also observed in the regional spectral power (Fig. S11). Moreover, group differences in regional dPTE and relative power were correlated with each other especially for the alpha band (Fig. S12). These results suggest a complex relationship between spectral power and PTE measures.

6.5. Comparison of scalar measures as potential biomarkers

In the Supplementary section 6.1, we mentioned the alpha- and beta-band mean global |dPTE| have a potential to become a good scalar diagnostic measure for TLE. Here we compare them with the other scalar measures: mean global relative power and mean global ImCoh. ROC curve analysis was performed and the results are shown in Fig. S13. For the mean global relative power, their delta-theta- and beta-band measures exhibit good performance for TLE/HC discrimination. For the mean global ImCoh, its delta-theta-band quantity exhibits good performance.

Interestingly, the three scalar measures, mean global |dPTE|, relative power, and ImCoh were complementary to each other in terms of the frequency bands. This indicates that a combined use of total 9 scalar measures (the frequency bands $\times$ the three measures) may provide a biomarker with high TLE/HC discrimination performance.

7. Cortical brain regions in the Brainnetome Atlas

The Brainnetome Atlas (Fan et al., 2016) was utilized in our atlas-based MEG analysis. The atlas is composed of 210 cortical regions and 36 subcortical regions. We focused on 210 cortical brain regions among the total 246 brain regions. Here we shows the list of the 210 cortical brain regions and their abbreviations we used (see Fan et al. (2016) for details); Table 1 is for label IDs from 1 to 108, and Table 2 is for label IDs from 109 to 210. The 210 regions are grouped into 20 modules, SFG, MFG, IFG, OrG, PrG, PCL, STG, MTG, ITG, FuG, PhG, pSTS, SPL, IPL, Pcun, PoG, INS, CG, MVOcC, and LOcC. The 20 modules are furthermore grouped into 6 lobes of the brain: Frontal (F), Temporal (T), Parietal (P), Insular (I), Limbic (L), and Occipital (O) lobes.

References

Bechtold, B., 2016. Violin plots for matlab. Github Project, https://github.com/bastibe/Violinplot-Matlab, DOI: 10.5281/zenodo.4559847 .

Campbell, R., 2016. notboxplot. Github Project, https://github.com/raacampbell/notBoxPlot .

Fan, L., Li, H., Zhuo, J., Zhang, Y., Wang, J., Chen, L., Yang, Z., Chu, C., Xie, S., Laird, A.R., et al., 2016. The human brainnetome atlas: a new brain atlas based on connectional architecture. Cerebral cortex 26, 3508–3526.

Guggisberg, A.G., Honma, S.M., Findlay, A.M., Dalal, S.S., Kirsch, H.E., Berger, M.S., Nagarajan, S.S., 2008. Mapping functional connectivity in patients with brain lesions. Annals of neurology 63, 193–203.

Haller, M., Donoghue, T., Peterson, E., Varma, P., Sebastian, P., Gao, R., Noto, T., Knight, R.T., Shestyuk, A., Voytek, B., 2018. Parameterizing neural power spectra. BioRxiv , 299859.

Hillebrand, A., Barnes, G.R., Bosboom, J.L., Berendse, H.W., Stam, C.J., 2012. Frequency-dependent functional connectivity within resting-state networks: an atlas-based meg beamformer solution. Neuroimage 59, 3909–3921.

Mahjoory, K., Schoffelen, J.M., Keitel, A., Gross, J., 2020. The frequency gradient of human resting-state brain oscillations follows cortical hierarchies. eLife 9, e53715.

Nolte, G., Bai, O., Wheaton, L., Mari, Z., Vorbach, S., Hallett, M., 2004. Identifying true brain interaction from eeg data using the imaginary part of coherency. Clinical neurophysiology 115, 2292–2307.

Qin, G., Hotilovac, L., 2008. Comparison of non-parametric confidence intervals for the area under the roc curve of a continuous-scale diagnostic test. Statistical Methods in Medical Research 17, 207–221.

Ranasinghe, K.G., Cha, J., Iaccarino, L., Hinkley, L.B., Beagle, A.J., Pham, J., Jagust, W.J., Miller, B.L., Rankin, K.P., Rabinovici, G.D., et al., 2020. Neurophysiological signatures in alzheimer's disease are distinctly associated with tau, amyloid-β accumulation, and cognitive decline. Science translational medicine 12.

Rubinov, M., Sporns, O., 2010. Complex network measures of brain connectivity: uses and interpretations. Neuroimage 52, 1059–1069.

Table 1: Brain regions and their abbreviations in the Brainnetome Atlas (ID: 1-108). Abbreviations: lh, left hemisphere; rh, right hemisphere; MNI, Montreal Neurological Institute coordinates.

lh.ID	rh.ID	Module	Abbreviation	Regions	lh.MNI	rh.MNI
1	2	Superior Frontal Gyrus	SFG A8m	medial area 8	-5, 15, 54	7, 16, 54
3	4		SFG A8dl	dorsolateral area 8	-18, 24, 53	22, 26, 51
5	6		SFG A9l	lateral area 9	-11, 49, 40	13, 48, 40
7	8		SFG A6dl	dorsolateral area 6	-18, -1, 65	20, 4, 64
9	10		SFG A6m	medial area 6	-6, -5, 58	7, -4, 60
11	12		SFG A9m	medial area 9	-5, 36, 38	6, 38, 35
13	14		SFG A10m	medial area 10	-8, 56, 15	8, 58, 13
15	16	Middle Frontal Gyrus	MFG A9/46d	dorsal area 9/46	-27, 43, 31	30, 37, 36
17	18		MFG IFJ	inferior frontal junction	-42, 13, 36	42, 11, 39
19	20		MFG A46	area 46	-28, 56, 12	28, 55, 17
21	22		MFG A9/46v	ventral area 9/46	-41, 41, 16	42, 44, 14
23	24		MFG A8vl	ventrolateral area 8	-33, 23, 45	42, 27, 39
25	26		MFG A6vl	ventrolateral area 6	-32, 4, 55	34, 8, 54
27	28		MFG A10l	lateral area10	-26, 60, -6	25, 61, -4
29	30	Inferior Frontal Gyrus	IFG A44d	dorsal area 44	-46, 13, 24	45, 16, 25
31	32		IFG IFS	inferior frontal sulcus	-47, 32, 14	48, 35, 13
33	34		IFG A45c	caudal area 45	-53, 23, 11	54, 24, 12
35	36		IFG A45r	rostral area 45	-49, 36, -3	51, 36, -1
37	38		IFG A44op	opercular area 44	-39, 23, 4	42, 22, 3
39	40		IFG A44v	ventral area 44	-52, 13, 6	54, 14, 11
41	42	Orbital Gyrus	OrG A14m	medial area 14	-7, 54, -7	6, 47, -7
43	44		OrG A12/47o	orbital area 12/47	-36, 33, -16	40, 39, -14
45	46		OrG A11l	lateral area 11	-23, 38, -18	23, 36, -18
47	48		OrG A11m	medial area 11	-6, 52, -19	6, 57, -16
49	50		OrG A13	area 13	-10, 18, -19	9, 20, -19
51	52		OrG A12/47l	lateral area 12/47	-41, 32, -9	42, 31, -9
53	54	Precentral Gyrus	PrG A4hf	area 4 (head and face region)	-49, -8, 39	55, -2, 33
55	56		PrG A6cdl	caudal dorsolateral area 6	-32, -9, 58	33, -7, 57
57	58		PrG A4ul	area 4 (upper limb region)	-26, -25, 63	34, -19, 59
59	60		PrG A4t	area 4 (trunk region)	-13, -20, 73	15, -22, 71
61	62		PrG A4tl	area 4 (tongue and larynx region)	-52, 0, 8	54, 4, 9
63	64		PrG A6cvl	caudal ventrolateral area 6	-49, 5, 30	51, 7, 30
65	66		PCL A1/2/3ll	area 1/2/3 (lower limb region)	-8, -38, 58	10, -34, 54
67	68		PCL A4ll	area 4 (lower limb region)	-4, -23, 61	5, -21, 61
69	70	Superior Temporal Gyrus	STG A38m	medial area 38	-32, 14, -34	31, 15, -34
71	72		STG A41/42	area 41/42	-54, -32, 12	54, -24, 11
73	74		STG TE1.0 and TE1.2	TE1.0/TE1.2	-50, -11, 1	51, -4, -1
75	76		STG A22c	caudal area 22	-62, -33, 7	66, -20, 6
77	78		STG A38l	lateral area 38	-45, 11, -20	47, 12, -20
79	80		STG A22r	rostral area 22	-55, -3, -10	56, -12, -5
81	82	Middle Temporal Gyrus	MTGs A21c	caudal area 21	-65, -30, -12	65, -29, -13
83	84		MTGs A21r	rostral area 21	-53, 2, -30	51, 6, -32
85	86		MTGs A37dl	dorsolateral area37	-59, -58, 4	60, -53, 3
87	88		MTGs aSTS	anterior superior temporal sulcus	-58, -20, -9	58, -16, -10
89	90	Inferior Temporal Gyrus	ITG A20iv	intermediate ventral area 20	-45, -26, -27	46, -14, -33
91	92		ITG A37elv	extreme lateroventral area37	-51, -57, -15	53, -52, -18
93	94		ITG A20r	rostral area 20	-43, -2, -41	40, 0, -43
95	96		ITG A20il	intermediate lateral area 20	-56, -16, -28	55, -11, -32
97	98		ITG A37vl	ventrolateral area 37	-55, -60, -6	54, -57, -8
99	100		ITG A20cl	caudolateral of area 20	-59, -42, -16	61, -40, -17
101	102		ITG A20cv	caudoventral of area 20	-55, -31, -27	54, -31, -26
103	104	Fusiform Gyrus	FuG A20rv	rostroventral area 20	-33, -16, -32	33, -15, -34
105	106		FuG A37mv	medioventral area37	-31, -64, -14	31, -62, -14
107	108		FuG A37lv	lateroventral area37	-42, -51, -17	43, -49, -19

Table 2: Brain regions and their abbreviations in the Brainnetome Atlas (ID: 109-210). Abbreviations: lh, left hemisphere; rh, right hemisphere; MNI, Montreal Neurological Institute coordinates.

lh.ID	rh.ID	Module	Abbreviation	Regions	lh.MNI	rh.MNI
109	110	Parahippocampal Gyrus	PhG A35/36r	rostral area 35/36	-27, -7, -34	28, -8, -33
111	112		PhG A35/36c	caudal area 35/36	-25, -25, -26	26, -23, -27
113	114		PhG TL	area TL (lateral posterior parahippocampal gyrus)	-28, -32, -18	30, -30, -18
115	116		PhG A28/34	area 28/34 (EC entorhinal cortex)	-19, -12, -30	19, -10, -30
117	118		PhG TI	area TI (temporal agranular insular cortex)	-23, 2, -32	22, 1, -36
119	120		PhG TH	area TH (medial PPHC)	-17, -39, -10	19, -36, -11
121	122	posterior Superior Temporal Sulcus	pSTS rpSTS	rostroposterior superior temporal sulcus	-54, -40, 4	53, -37, 3
123	124		pSTS cpSTS	caudoposterior superior temporal sulcus	-52, -50, 11	57, -40, 12
125	126	Superior Parietal Lobule	SPL A7r	rostral area 7	-16, -60, 63	19, -57, 65
127	128		SPL A7c	caudal area 7	-15, -71, 52	19, -69, 54
129	130		SPL A5l	lateral area 5	-33, -47, 50	35, -42, 54
131	132		SPL A7pc	postcentral area 7	-22, -47, 65	23, -43, 67
133	134		SPL A7ip	intraparietal area 7 (hIP3)	-27, -59, 54	31, -54, 53
135	136	Inferior Parietal Lobule	IPL A39c	caudal area 39 (PGp)	-34, -80, 29	45, -71, 20
137	138		IPL A39rd	rostrodorsal area 39 (Hip3)	-38, -61, 46	39, -65, 44
139	140		IPL A40rd	rostrodorsal area 40 (PFt)	-51, -33, 42	47, -35, 45
141	142		IPL A40c	caudal area 40 (PFm)	-56, -49, 38	57, -44, 38
143	144		IPL A39rv	rostroventral area 39 (PGa)	-47, -65, 26	53, -54, 25
145	146		IPL A40rv	rostroventral area 40 (PFop)	-53, -31, 23	55, -26, 26
147	148	Precuneus	PcunA7m	medial area 7 (PEp)	-5, -63, 51	6, -65, 51
149	150		PcunA5m	medial area 5 (PEm)	-8, -47, 57	7, -47, 58
151	152		PcundmPOS	dorsomedial parietooccipital sulcus (PEr)	-12, -67, 25	16, -64, 25
153	154		PcunA31	area 31 (Lc1)	-6, -55, 34	6, -54, 35
155	156	Postcentral Gyrus	PoG A1/2/3ulhf	area 1/2/3 (upper limb, head and face region)	-50, -16, 43	50, -14, 44
157	158		PoG A1/2/3tonIa	area 1/2/3 (tongue and larynx region)	-56, -14, 16	56, -10, 15
159	160		PoG A2	area 2	-46, -30, 50	48, -24, 48
161	162		PoG A1/2/3tru	area1/2/3 (trunk region)	-21, -35, 68	20, -33, 69
163	164	Insular Gyrus	INS G	hypergranular insula	-36, -20, 10	37, -18, 8
165	166		INS vIa	ventral agranular insula	-32, 14, -13	33, 14, -13
167	168		INS dIa	dorsal agranular insula	-34, 18, 1	36, 18, 1
169	170		INS vId/vIg	ventral dysgranular and granular insula	-38, -4, -9	39, -2, -9
171	172		INS dIg	dorsal granular insula	-38, -8, 8	39, -7, 8
173	174		INS dId	dorsal dysgranular insula	-38, 5, 5	38, 5, 5
175	176	Cingulate Gyrus	CG A23d	dorsal area 23	-4, -39, 31	4, -37, 32
177	178		CG A24rv	rostroventral area 24	-3, 8, 25	5, 22, 12
179	180		CG A32p	pregenual area 32	-6, 34, 21	5, 28, 27
181	182		CG A23v	ventral area 23	-8, -47, 10	9, -44, 11
183	184		CG A24cd	caudodorsal area 24	-5, 7, 37	4, 6, 38
185	186		CG A23c	caudal area 23	-7, -23, 41	6, -20, 40
187	188		CG A32sg	subgenual area 32	-4, 39, -2	5, 41, 6
189	190	MedioVentral Occipital Cortex	MVOcC cLinG	caudal lingual gyrus	-11, -82, -11	10, -85, -9
191	192		MVOcC rCunG	rostral cuneus gyrus	-5, -81, 10	7, -76, 11
193	194		MVOcC cCunG	caudal cuneus gyrus	-6, -94, 1	8, -90, 12
195	196		MVOcC rLinG	rostral lingual gyrus	-17, -60, -6	18, -60, -7
197	198		MVOcC vmPOS	ventromedial parietooccipital sulcus	-13, -68, 12	15, -63, 12
199	200	Lateral Occipital Cortex	LOcC mOccG	middle occipital gyrus	-31, -89, 11	34, -86, 11
201	202		LOcC V5/MT+	area V5/MT+	-46, -74, 3	48, -70, -1
203	204		LOcC OPC	occipital polar cortex	-18, -99, 2	22, -97, 4
205	206		LOcC iOccG	inferior occipital gyrus	-30, -88, -12	32, -85, -12
207	208		LOcC msOccG	medial superior occipital gyrus	-11, -88, 31	16, -85, 34
209	210		LOcC lsOccG	lateral superior occipital gyrus	-22, -77, 36	29, -75, 36
